# Spectral Analysis of Extrahepatic Bile Ducts During Normothermic Liver Machine Perfusion

**DOI:** 10.3390/bioengineering12090966

**Published:** 2025-09-09

**Authors:** Philipp Zelger, Benjamin Jenewein, Magdalena Sovago, Felix J. Krendl, Andras T. Meszaros, Benno Cardini, Philipp Gehwolf, Johannes D. Pallua, Simone Graf, Stefan Schneeberger, Margot Fodor, Rupert Oberhuber

**Affiliations:** 1 Department for Hearing, Speech, and Voice Disorders, Medical University of Innsbruck, Anichstraße 35, 6020 Innsbruck, Austria; philipp.zelger@i-med.ac.at (P.Z.); simone.graf@i-med.ac.at (S.G.); 2 Department of Visceral, Transplant and Thoracic Surgery, OrganLifeTM, Medical University of Innsbruck, Anichstraße 35, 6020 Innsbruck, Austria; benjamin.jenewein@student.i-med.ac.at (B.J.); magdalena.sovago@student.i-med.ac.at (M.S.); felix.krendl@i-med.ac.at (F.J.K.); andras.meszaros@i-med.ac.at (A.T.M.); benno.cardini@i-med.ac.at (B.C.); philipp.gehwolf@i-med.ac.at (P.G.); stefan.schneeberger@i-med.ac.at (S.S.); 3 Department for Orthopaedics and Traumatology, Medical University of Innsbruck, Anichstraße 35, 6020 Innsbruck, Austria; johannes.pallua@i-med.ac.at

**Keywords:** normothermic liver machine perfusion, bile ducts, liver transplantation, hyperspectral imaging

## Abstract

**Background**: Biliary complications (BC) affect 5–32% of liver transplant (LT) patients and include strictures, leaks, stones, and disease recurrence. Their risk increases with extended criteria donor (ECD) livers, contributing to early graft dysfunction. Normothermic liver machine perfusion (NLMP) helps reduce bile duct (BD) damage overall, but anastomotic region issues persist. This study assessed hyperspectral imaging (HSI) as a non-invasive method to evaluate BD viability during NLMP. **Methods**: Eleven donor livers underwent NLMP with HSI at the start and end. Seven were transplanted; four were discarded. HSI measured tissue oxygenation, perfusion, and composition. The spectral data were analyzed using ANOVA, post hoc t-tests, and multifactorial ANOVA to assess spectral changes related to BD position, transplant status, and occurrence of BC. **Results**: Significant spectral changes were found in the BD region during NLMP. Transplanted livers that developed BC showed changes between 525 and 850 nm, while discarded ones had changes between 625 and 725 nm. Specific spectral bands (500–575 nm, 775–1000 nm) were linked to transplant outcomes and BC. **Conclusions:** HSI shows promise as a non-invasive tool to assess BD viability during NLMP and may help predict post-transplant BC.

## 1. Introduction

Biliary complications (BC) continue to be a significant cause of morbidity after liver transplantation (LT). They occur in 5–32% [1,2] and include strictures (anastomotic and non-anastomotic), leaks, stones, and the recurrence of primary biliary diseases [1].

Since LT has been established as a successful treatment for end-stage liver disease, organ shortage has become a major challenge. To bridge the organ supply–demand gap, the use of livers from extended criteria donors (ECD) has steadily increased during recent decades [3]. Transplantation of more marginal organs poses the risk of higher rates of early allograft dysfunction (EAD), primary non-function (PNF), and BC [4,5,6,7,8,9].

While static cold storage has been the gold standard in terms of organ preservation since the late 1960s, as the donor pool has shifted towards more marginal the need for improved preservation methods has resurfaced. Normothermic liver machine perfusion (NLMP) has gained renewed interest as it provides a platform for viability assessment leading to increased organ utilization and potentially decreased downstream effects of ischemia–reperfusion injury in the recipient (Perfusing the liver before transplantation has resulted in the attenuation of ischemia-induced biliary damage and the ability to safely test bile duct (BD) viability) [10,11,12,13].

NLMP involves ex situ perfusion of the graft with an oxygenated, nutrient-enriched, erythrocyte-based perfusate at 37 °C [6,14].

We have recently demonstrated that the rate of non-anastomotic strictures (NAS) was lower after NLMP when compared to static cold storage livers [6]. However, distal BD and anastomotic complications were unchanged. This phenomenon has been attributed to the bile production and bile composition-based selection of organs before transplantation, since the analysis of bile would yield biomarkers indicative of the condition of the biliary tree rather than the most distal segment of the main BD. A further study performed at our department aimed to propose a qualitative and quantitative assessment of the distal BD during transplantation to test the hypothesis that anastomotic (region)—related complications can be predicted using hyperspectral imaging (HSI). HSI allows for a contactless, real-time quantitative evaluation of tissue oxygenation, micro-perfusion, and organ hemoglobin and water concentration [15,16,17,18,19,20,21,22,23,24,25]. In a second step, tissue-specific features were extracted from HSI data and elaborated through convolutional neural networks (CNNs) [13]. The CNN-based analysis yielded a correct classification in 72% and 69% for BC/no BC. Combining HSI data with donor and recipient factors led to an increased accuracy of 94% in predicting BC. Thus, this novel approach represents a non-invasive technique for predicting postoperative BC. However, an apparent limitation of this study was that measurements were applied after completion of the biliary anastomosis; consequently, intraoperative interventions were not intended. Building on these preliminary data, a prospective study using a similar algorithm during NLMP has been started to design a real-time diagnostic tool capable of predicting and limiting BC [13].

As an emerging optical technique, HSI is used in disease diagnosis and image-guided surgery to produce quantitative diagnostic information about tissue pathology, morphology, and chemical composition.

HSI is a technique that leverages distinct spectral signatures, akin to “fingerprints,” to analyze the spatial distribution of chemical compounds [26]. These spectral fingerprints enable the identification and characterization of compositionally distinct substances based on their chemical properties [27,28,29,30,31,32]. HSI finds extensive applications in diverse research fields, including geology, botany [33,34,35,36], the food industry, art preservation, and biomedical science [37,38,39,40,41,42,43,44,45,46], and is crucial in intraoperative imaging in biomedical science, addressing specific surgical challenges and forensic analysis [47,48,49,50,51,52,53,54,55,56,57,58,59]. HSI acquires data across the visible (VIS = 400–650 nm) and near-infrared (NIR = 750–1000 nm) regions of the electromagnetic spectrum. This technique was also applied intraoperatively to assess the capability of discriminating biliary anatomy from surrounding biological tissue [18,25]. Absorbance spectra measured from BD, gall bladder, and liver showed a dependence on tissue composition and bile concentration, with agreement between human and porcine datasets [60].

While there have been recent advancements in biliary visualization methods, such as fluorescent intraoperative cholangiography [61], these methods still require the administration of exogenous fluorescent dyes. Recently, HSI data from human LT procedures and porcine model operations were analyzed and compared to discriminate biliary structures from surrounding tissue. For this purpose, chromophores present in bile fluid and tissue composition were explored as the cause of characteristic absorbances observed in recorded HSI spectra of liver, gall bladder, and BD [12]. Despite promising advances, most studies have been conducted on relatively small sample sizes, which limits the generalizability of their findings. Long-term follow-up data on BC and graft outcomes are often lacking, making it difficult to fully assess the clinical utility of these techniques. Technical challenges, such as variable intraoperative lighting conditions and motion artifacts, continue to affect the reliability of HSI in real-time surgical environments, despite ongoing efforts to develop calibration methods. Finally, many machine learning models developed to analyze spectral data have yet to undergo rigorous external validation, limiting their immediate translation into routine clinical practice. The relation between different HSI spectra on extrahepatic BD and their dynamic changes over NLMP has not been studied in a clinical setting. Moreover, formal evidence that characteristic absorbances change over NLMP and may offer additional information on BD viability has never been provided so far.

This study aims to investigate whether HSI is suitable to detect differences in the reflection spectra of BD, over the time course of NLMP for transplanted and not transplanted organs, as well as differences in spectra of BD between organs developing BC and those that do not develop a BC.

## 2. Materials and Methods

### 2.1. Study Population

Eleven donors’ livers were enrolled in this study between March 2023 and October 2023. All livers were subjected to NLMP. Based on established center-specific viability criteria [3], seven livers were subsequently transplanted, while four organs were discarded. An overview summarizing essential characteristics of donors, recipients, liver allografts, MP times, and the postoperative outcome is presented in Table 1. Prior to procurement, all livers were flushed in situ using either histidine-tryptophan-ketoglutarate (HTK) or Institut Georges Lopez-1 (IGL-1) preservation solution. Upon arrival at the transplant center, livers were prepared on the back table for NLMP. A standardized perfusion and assessment protocol was applied, including blood gas analysis and biochemical evaluation of perfusate samples to assess liver function [3].

### 2.2. Study Procedure

The local ethics committee approved the study (EK 1389/2022), and the study was conducted according to the principles of the Declaration of Helsinki.

During the transplant procedure, the resected segment of the donor bile duct (BD) was collected upon reconstruction and analyzed using real-time live confocal microscopy (RTCM) as well as conventional histopathology. After trimming the BD to remove malperfused tissue and completing the biliary anastomosis, hyperspectral imaging (HSI) measurements were performed both in situ during liver transplantation (LT) and on the back table, in order to evaluate the perfusion and oxygenation status of the regions of interest (ROI).

The primary postoperative endpoints were biliary anastomotic strictures (AS) and biliary leakages. BD imaging following LT was conducted using computed tomography (CT) or magnetic resonance cholangiopancreatography (MRCP), typically in response to suspected graft dysfunction indicated by elevated liver function parameters or clinical signs of graft-related deterioration [6]. AS was defined as a significant narrowing of the biliary tree at the anastomotic site, with or without upstream biliary dilatation visible on MRCP. In cases where narrowing at the anastomosis was detected by endoscopic retrograde cholangiopancreatography (ERCP) and/or percutaneous transhepatic cholangiography (PTC), the lesion was classified as a cholangiographic stricture if it required intervention such as balloon dilation and/or stent placement.

Biliary leakage was defined by elevated bilirubin level (>3 times the serum level) in abdominal fluid collections. Leakage required radiological intervention or re-laparotomy in all cases [62].

### 2.3. Normothermic Liver Machine Perfusion—Centre-Specific Protocol

The decision to apply NLMP at our center was based on one or more of the following indications: (I) uncertain organ quality, (II) complex recipient, and (III) logistics. According to local protocol, MP was performed using the OrganOx metra^®^ system [3], and the perfusion time on the OrganOx metra^®^ system depended on the time required for assessment, decision-making, and logistics. The decision to discard or transplant an organ was based on the evaluation of key quality parameters, namely the preservation of physiological pH values (7.3–7.45) without sodium bicarbonate supplementation after 2 h of NLMP; a prompt decline and maintenance of lactate to physiological values (≤18 mg/dL), as well as bile output and biliary pH > 7.4 [3,63]. These indicators are considered to reflect appropriate organ function. Furthermore, elevated aspartate aminotransferase (AST), alanine aminotransferase (ALT) (>20,000), and lactate dehydrogenase (>20,000) levels indicate a need for caution [3,4].

### 2.4. Hyperspectral Imaging of Extrahepatic Bile Ducts During Normothermic Liver Machine Perfusion

**Figure 1:**
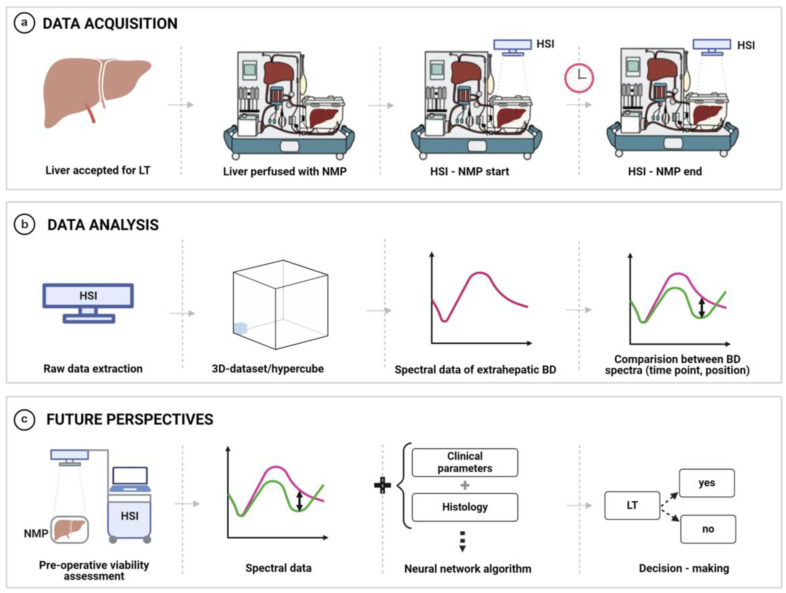
HSI measurements and data analysis process. (**a**) HSI measurement setup. (**b**) Data acquisition process. (**c**) Future perspectives and possible clinical applications.

A contactless and non-ionizing radiation imaging system (TIVITA^®^ Tissue System, Diaspective Vision GmbH, Am Salzhaff, Germany (DE)) was used to acquire HSI data, applying standardized conditions according to previously reported settings [18,19,64,65]. The HSI camera was placed 50 cm above a region of interest (ROI), at the beginning (15 min after starting NLMP) and at the end of NLMP (Figure 1a), at a room temperature of 20–25 °C in the operating room. HSI is capable of studying spectra up to 0.6 cm deep into the tissue. All ambient light was turned off during the HSI acquisition to avoid artefacts. The ROI included the cannulated extrahepatic BD (0.5 cm^2^ area). The illumination of the BD was performed using six spectral ranges from 500 to 1000 nm. Effective pixels were 640 × 480 (x-, y-axis).

Two HSI measurements were carried out on each sample, as illustrated in Figure 1.

The software (TIVITA Suite Tissue) provides a red-green-blue (RGB) image and four false-colour images illustrating physiologic parameters of the recorded tissue area, which quantifies values of the parameters from blue (low values) to red (high values).

Given its contactless and rapid applicability, HSI measurements did not interfere with the perfusion procedure (about 10 s for recording and the near-real-time option of visualization and interpretation). Acquired RGB and color images were collected and stored for further analysis. Segmentation of the raw HSI spectra and determining ROIs along the BD, divided into distal and proximal regions using visual inspection, image labelling, and outlining by an experienced surgeon. The process of HSI measurements and data analysis is summarized in Figure 1.

### 2.5. Definitions

#### 2.5.1. Graft Loss and Graft Dysfunction

Graft loss was defined as either patient death or the need for retransplantation (i.e., a second re-LT). Primary non-function (PNF) was defined as a peak AST level ≥ 3000 IU/L, in combination with at least one of the following criteria measured on postoperative day 3 (excluding cases of biliary obstruction): INR ≥ 2.5, serum lactate ≥ 4 mmol/L, or total bilirubin ≥ 10 mg/dL [66]. Early allograft dysfunction (EAD) was defined according to the Olthoff criteria [67].

#### 2.5.2. Rejections

Rejection episodes were diagnosed based on clinical suspicion and confirmed by liver biopsy. In cases of suspected rejection, patients were treated with an intravenous steroid pulse consisting of 500 mg of methylprednisolone daily for three consecutive days, followed by an escalation of maintenance immunosuppression.

#### 2.5.3. Infectious Complications and Sepsis

Any documented infection requiring antimicrobial treatment was recorded as an infectious complication. Sepsis was defined as a life-threatening organ dysfunction caused by a dysregulated host response to infection, following the third international consensus definition for sepsis and septic shock [68].

#### 2.5.4. Balance of Risk (BAR) Score

The BAR score incorporates six variables (MELD score, donor age, recipient age, cold ischemia time, re-transplantation, and the need for life support) available at the time of organ acceptance, ranging from 0 to 27 points. BAR score values have been calculated according to the publication by Dutkowski et al. [69] using the online BAR score calculator.

#### 2.5.5. Classification and Quantification of Complications

Postoperative complications were graded according to the Clavien-Dindo classification system [70]. Clavien-Dindo grades I and II were recorded as minor complications. Clavien-Dindo Grade IIIa complications were considered moderate complications, while Grade IIIb or higher complications were defined as significant. Complications were further quantified using the comprehensive complication index (CCI) within 3 months and 1 year after transplantation [70,71].

### 2.6. Spectral Analysis and Data Processing

The segmentation of the raw HSI spectra has already been demonstrated [18,72,73,74,75].

Depending on the dataset’s different approaches, ROIs can be determined by visual inspection and labelling of acquired images using semi-automated tools based on spectral angle mapper algorithms [76]. In this work, the ROIs have been outlined by an experienced surgeon. The ROI has subsequently been divided into three regions along the BD: distal, medial, and proximal.

### 2.7. Statistical Analysis

Each single spectrum (i.e., the pixels of the hyperspectral image) has been normalized before the analysis. The data is presented as mean spectra with standard deviation. For further analysis, the spectral data was analyzed using a rolling average. Here, the spectra data underwent a moving average mean calculation with a window size of 5 wavenumbers to reduce noise in the data. This averaged data was subsequently analyzed, independently for all averaging spectral steps, using a multifactorial ANOVA with the position and appearance of the complication factor for the transplanted organs and with the factors position and transplanted vs. not transplanted organs for all the datasets. For significant results, a post hoc *t*-test was calculated. For the comparison between BD complication vs. no complications and transplanted vs. not transplanted the effect size estimation using Cohen’s d was calculated. Sphericity was tested using Mauchly’s Test of Sphericity.

Data are presented as medians with interquartile ranges (IQR). Comparative analysis of donor parameters and operative outcomes in the BC and non-BC groups was conducted using the Chi-square and Fisher’s exact test for categorical variables and the Mann–Whitney-U-Wilcoxon test for continuous variables. Ordinal variables were treated as continuous variables. Two-tailed *p*-values < 0.05 were considered significant throughout the entire analysis. Statistical analysis was performed using SPSS Statistics Version 27.0 for Macintosh (IBM Corporation, Armonk, NY, USA) and GraphPad Prism 9 for macOS version 9.3.1.3.

## 3. Results

### 3.1. Recipient, Donor, and Preservation Characteristics

Seven consecutive deceased donor livers were included in the study. The median donor age was 61 years (51–68 years). Cold ischemia time (CIT) was 6 h (5–8 h). Per centre preference and routine, all seven livers were preserved by NLMP and assessed before transplantation. In most cases, the indication for NLMP use was not driven by a single factor but rather by a combination of multiple factors. NLMP was applied for marginal donors in 43% (three livers), complex recipients in 14% (one liver), and logistics in 57% (four livers).

All transplantations except one were performed during daytime hours. The median NLMP time was 19 h (11–21 h). The total preservation time was 22 h (18–27 h). One (14%) graft was retrieved from donation after cardiac death (DCD) donors (Maastricht category III); the remaining grafts stemmed from donation after brain death (DBD) donors. Median recipient MELD and BAR scores were 16 (13–19 h) and 7 (5–8 h), respectively. The median recipient age was 59 years (58–65 years). Five (71%) donors were male, and 2 (29%) were female.

The median ICU and entire hospital stay were 3 (3–5 h) and 16 days (15–26 h), respectively. Three patients (43%) developed EAD. Clavien-Dindo grade ≥3 complications occurred in five (71%) of 7 patients. Arterial complications occurred in three (43%) patients (two anastomotic stenosis and one arterial defect in the jump graft). Early (≤30 days) BC was detected in two (29%) patients who developed an AS. These were not clinically relevant (bleeding, cholangitis) and were detected based on rising cholestatic parameters without specific symptoms. Both AS were treated by ERCP. One patient (14%) developed a biliary leak as a late (>30 days) BC, which was treated by surgery. No patients developed NAS, post-transplant cholangiopathy, or PNF. One patient developed an acute rejection based on clinical suspicion (not biopsy proven) and was treated with an intravenous steroid pulse of 500 mg methylprednisolone for 3 days, followed by an increase in maintenance immunosuppression. In five patients, an infection requiring antimicrobial treatment was recorded, but none developed sepsis. The unplanned readmission rate within 30 days was 14% (one patient). No patients were listed for re-transplantation. No patient died during the 6-month-long follow-up period. Recipient, donor demographics, and postoperative outcome parameters are described in Table 1. NLMP hepatic artery and portal vein flows were >150 mL/min and >500 mL/min for all livers over the entire course.

### 3.2. Spectral Analysis

**Figure 2:**
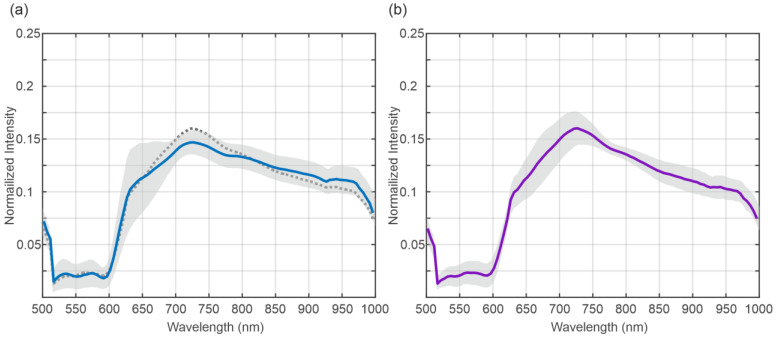
Mean spectra of the biliary ducts at the beginning (blue) (**a**) and at the end (purple) (**b**) of normothermic machine perfusion for transplanted organs. The grey shaded area indicates the standard deviation of the data. The dotted line in (**a**) indicates the mean spectra at the end of normothermic machine perfusion.

The mean spectra of the BD of the transplanted organs are shown in Figure 2. Figure 2a shows the mean spectra at the beginning, and Figure 2b shows the mean spectra at the end of NLMP. The grey area indicates the standard deviation of the data. The dashed line in Figure 2a indicates the mean value of the data presented in Figure 2b for comparison. The visual comparison of the mean spectra reveals only a small difference between the two time points.

**Figure 3:**
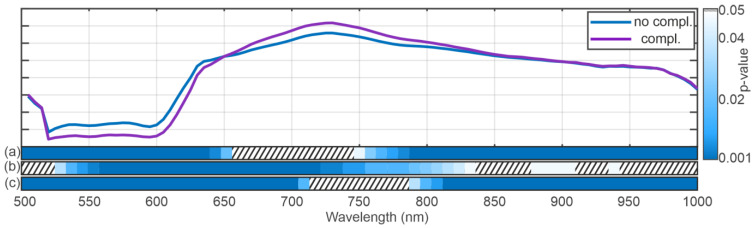
The mean spectrum from the biliary duct shows no complications (blue) and exhibits complications (purple). The blue-shaded areas below the spectra correspond to the results of the running ANOVA. Here, the dashed area indicates non-significant (*p* > 0.05) results. The blue shaded area indicates the *p*-value as described by the color bar on the right. The figure shows the results for the ANOVA with the factors position (a), presence of a biliary duct complication (b), and the recording time (c).

Figure 3 shows the results of the running ANOVA for the factor’s position (distal vs. proximal), (a), the appearance of complications, (b), and the time of recording (beginning vs. end of NLMP), (c), below mean spectra from BD showing no complications (blue) and exhibiting complications (purple). The colour code represents the resulting *p*-value as indicated by the colour bar on the right of the plot. The dashed areas show spectral regions where no significant difference in the spectra has been revealed. The ANOVA results indicate highly significant changes in the spectra for the position parameter in the spectral regions between 500 and 650 nm and between 750 and 1000 nm The analysis results regarding the complications (b) show that the whole spectral range exhibits significant differences in both categories, focusing on the region between 525 and 825 The time of recording term reveals a significant result everywhere besides the region between around 725 and 775 nm.

The effect size analysis using Cohan’s d, for the comparison between BD complication vs. no complication, exceeded 0.2 (small effect) between approximately 500 nm and 650 nm. Outside this range, d dropped below 0.2 and power correspondingly fell, demonstrating reduced ability to detect group differences (Figure 1).

**Figure 4:**
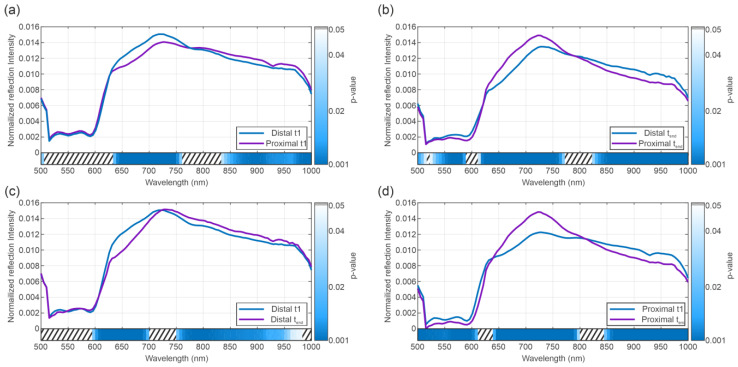
Post hoc *t*-test between single modalities. Comparison between distal and proximal biliary duct spectra at the beginning of the NLMP (**a**), at the end of the NLMP (**b**). Comparison between the spectra at the beginning and the end of the NLMP for the distal (**c**) and proximal (**d**) part of the BD.

The results of the post hoc *t*-test according to the position and time point of the spectra are shown in Figure 4a–d. Here, the data exhibits highly significant results for all four comparisons. The figure shows the results for pairwise running t-tests for the groups “distal BD beginning” vs. “proximal BD beginning” (a), “distal BD end” vs. “proximal BD end” (b), “distal BD beginning” vs. “distal BC end” (c), “proximal NLMP beginning” vs. “proximal BD end” (d).

**Figure 5:**
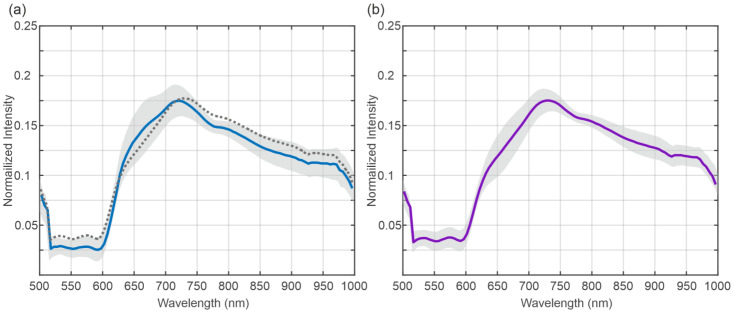
Mean spectra of the biliary ducts at the beginning (blue) (**a**) and at the end (purple) (**b**) of normothermic machine perfusion for non-transplanted organs. The grey shaded area indicates the standard deviation of the data. The dotted line in (**a**) indicates the mean spectra at the end of normothermic machine perfusion.

Figure 5 presents the mean spectra of the organs that were not transplanted. Figure 5a shows the mean spectra (blue) and the standard deviation (grey) of the spectra recorded at the beginning of the NLMP, and Figure 5b shows the respective spectral data at the end of the NLMP. Figure 5a includes the mean spectra of the data at the end of the NLMP as a comparison (dashed line). The mean spectra exhibit a bigger deviation during the NLMP for the not-transplanted organs, in contrast to transplanted organs (Figure 2a). This change is most prominent between 625 and 725 nm.

The results of an effect size analysis using Cohan’s d for the transplanted vs. non-transplanted data show a large effect (>0.8) for the wavelength 500 to 600 nm and a medium effect (>0.5) in the spectral regions between 600 and 630 nm and in the region between 750 and 1000 nm.

**Figure 6:**
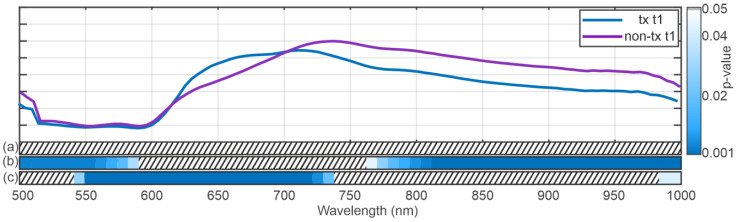
Mean spectrum of the transplanted (blue) and non-transplanted organs (purple) as an indicator for the wavelength regions. The figure shows the results for an ANOVA over all the datasets (transplanted and not transplanted organs) with the factors position (a), transplanted vs. not-transplanted (b), and the time of recording (c). Here, only the status “transplanted vs. non-transplanted”, and the time of comparison yield statistically significant results.

Figure 6 shows the results of the running ANOVA over all the datasets (transplanted and not transplanted organs) for the position of the factor (distal vs. proximal) (a), transplanted vs. not transplanted, (b) and the time of recording (c) below the mean spectra of the transplanted (blue) and non-transplanted organs (purple). Here, the factor position does not yield a significant result, whereas the factors transplanted vs. not transplanted (b) and time of recording (c) show significant results in some spectral regions. The spectral regions reaching a significant result for the comparison transplanted vs. not transplanted were found at the beginning at the end of the spectra. The statistically significant areas in the comparisons of transplanted vs. non-transplanted liver grafts and time of recording show an almost opposing pattern.

## 4. Discussion

Most risk factors for BC are non-modifiable. Therefore, it is crucial to focus on careful organ selection, minimizing cold ischemia time (CIT)—one of the few modifiable risk factors -, flushing bile ducts following organ retrieval and thoroughly assessing the organ during machine perfusion (MP) to reduce the overall risk of BC [77,78,79,80,81]. Additionally, ex situ graft preservation by NLMP may serve as a platform for the repair of pre-injured liver grafts before implantation [6,79,82]. A low bile pH (<7.4) during NLMP currently serves as the best predictor for ischemic-type bile duct lesions [83]. Bile bicarbonate represents a valid surrogate marker of cholangiocyte function [83,84,85,86]. Further techniques such as microRNA, metabolomics, and glycomics assessments have been recently evaluated [79]. In light of these developments, preoperative viability assessment techniques are warranted. As a non-invasive tool, HSI can provide additional intraoperative information on BD viability, offering an automated and standardized system [72]. A recent analysis demonstrated that deep learning-based modelling based on spectral information from HSI datasets might be used as a tool for non-invasive prediction of BC after LT [13].

The results of the present study indicate that using HSI in the context of NLMP allows for the detection of significant differences in the spectra of the extrahepatic BD between different classes of BD data based on potential biochemical and physical alterations in the BD tissue.

The comparison between the spectra at the beginning and the end of NLMP revealed a significant difference in the ANOVA analysis Figure 3c. The spectral regions in which this result occurred coincide with the significant regions of the analysis comparing the temporal component of the NLMP treatment. This suggests a correlation between the temporal change in spectra and the position of the bile duct (distal vs. proximal). The post hoc comparisons performed in Figure 4a–d confirmed this assumption. Comparing the spectra from BD in liver grafts of transplant patients with the incidence of postoperative BC, significant variations in the spectral regions between 525 nm and 850 nm (Figure 3b) were detected. Additionally, a comparison between transplanted and non-transplanted organs revealed further notable differences in the HSI spectra. Here, the significant regions were found between 500 and 575 nm and between 775 and 1000 nm (Figure 6b). This indicates an overlap in the statistically significant regions between 550 and 575 nm when comparing transplanted versus non-transplanted organs and the presence versus absence of BC. The spectral region between 500 and 575 nm is associated with the absorption characteristics of oxygenated hemoglobin (HbO_2_) and bile pigments, particularly biliverdin and bilirubin. These differences are likely due to variations in bile pigment concentration, as biliverdin, which absorbs strongly at 675 nm and may increase as a result of bile’s acidic environment during fasting or ischemic conditions, leading to bilirubin oxidation. The spectral changes detected in this region suggest that HSI can identify biochemical alterations in BD, potentially serving as early markers for BC. The presence of biliverdin and bilirubin may offer valuable insights into BD health and the risk of developing post-transplant cholangiopathy. The high-water content of bile (95%) and lipid presence is reflected in the spectral absorbance peaks at around 970 nm and 930 nm, respectively [87,88]. The findings of our study align with Cooney et al. [60], emphasizing these components’ role in the absorption spectra. The differences in this region highlight potential edema or necrotic processes in extrahepatic BD, especially in non-transplanted organs, where significant deviations were observed (Figure 6).

Another interesting observation was the inverse statistically significant regions in the central (575 to 775 nm) and high wavelength (775 to 1000 nm) regions when comparing BC and whether the organs were transplanted. It appears that the central region of the spectra showed a significant difference for the development of BC but not for distinguishing between transplanted and non-transplanted organs. In contrast, the high wavelength region exhibited the opposite relationship, i.e., significance for the difference between transplanted and non-transplanted but no significance for BC. In the 575–650 nm range, significant spectral differences were observed (BC vs. no BC, Figure 3, and transplanted vs. not transplanted livers, Figure 6). This may be associated with the absorption of HHb and collagen/elastin fibers, which are critical structural proteins in BD and surrounding tissues. Increased absorbance in this range, particularly in association with BC, may reflect deoxygenated hemoglobin accumulation and structural changes due to ischemic injury. In the near-infrared range, 650–750 nm, the primary absorptive components include water, Hb, and biliverdin, with spectral differences indicating variations in bile composition and tissue hydration between transplanted and non-transplanted organs, as well as between those with or without BC. This range should be valuable for assessing hydration status and tissue edema. The 750–850 nm range features strong absorption by collagen and elastin, with significant spectral changes observed in organs with post-transplant BC, suggesting structural damage from ischemia–reperfusion injury or other stressors. Finally, the 850–1000 nm range, dominated by water and lipid absorption, revealed significant differences in absorbance patterns, reflecting alterations in water and lipid content that may be due to bile stasis or necrotic changes (Figure 6). Monitoring this range is crucial for evaluating tissue composition, edema, and lipid metabolism, which are important for assessing BD health and function during LT. Biliverdin and bilirubin are critical in the bile’s absorbance characteristics, with biliverdin showing a strong peak at 675 nm [87,88]. Changes in this range reflect biochemical shifts in bile composition, particularly during ischemic conditions or preoperative fasting, leading to increased biliverdin levels due to bilirubin oxidation [60]. Collagen and elastin contribute significantly to the absorbance in this range. The study’s results suggest that changes in these proteins’ absorbance are linked to ischemic injury or structural integrity loss in the BD, providing essential clues for assessing transplant viability and the risk of complications [89].

A study by Cooney et al. [60] analyzed the absorption spectra of liver parenchyma, BD, and bile in human and pig livers using HSI. The authors demonstrated that liver parenchyma generally had higher absorption capacity than BD across most of the HSI-investigated spectral range (500–1000 nm), except around 930 nm. This higher absorption at 930 nm on BD is likely due to the high water (95% of bile composition) and lipid content in the bile-filled biliary structures as the gallbladder and BD (lipid absorption maxima: approximately 930–970 nm) [60]. Additionally, the study examined the absorption spectra of biliary structures, which are primarily influenced by the amount of biliverdin (a bile pigment with absorption maxima around 675 nm) and structural proteins like collagen and elastin. During periods of liver undersupply (such as preoperative fasting), bile acidity increases, and bile pH drops, altering bile composition. Notably, bilirubin can more easily oxidize to biliverdin in acidic conditions, leading to higher biliverdin levels. A drop in bile pH thus results in increased biliverdin. Concurrently, lipid concentrations in bile decrease with undersupply. These biochemical changes were detectable in the study through changes in the biliary absorption spectra using HSI. According to these findings, it might be inferred that biological changes in the BD due to a drop in Bile pH are likely visible in the 650–700 nm wavelength range during HSI [60], which aligns with the findings of our research. In our analysis, we observed the most relevant differences in the spectra of discarded livers, according to the factor time of NLMP, especially in the region between 600 nm and 725 nm (Figure 6). When comparing transplanted and discarded organs and the presence of BC in transplanted organs, the most structural spectral modification was only significant for the appearance of BC in this specific spectral region (Figure 6). Our results match those made by Cooney et al. and may be associated with a subsequent oxidation and necrotic process of extrahepatic BD [60].

An analysis by Felli et al. [72] identified the bile pigment biliverdin and the structural proteins collagen and elastin as contributors to the absorbance spectra of bile ducts and gall bladder, depending on fasting periods [72]. Similarly to the fasting periods (such as preoperative fasting) described in the work by Felli et al. [72], the CIT followed by normothermic perfusion in the context of NLMP might lead to detectable changes in the biliary absorption spectra.

In summary, this study confirms changes in the discussed spectral region by comparing the spectra at the beginning and at the end of NLMP. Additionally, we identified significant differences between the BD spectra of organs with and without postoperative BC, particularly in the wavelength range of 500–800 nm (Figure 3). These alterations may be justified due to (i) pre-existing biliary damage or (ii) BD undersupply after organ explant, which can lead to detectable biochemical changes during NLMP, such as decreased lipid concentration and increased biliverdin.

The relatively small cohort of this study represents an apparent limitation. Further, the limited potentiality of HSI to explore deeper tissue layers allows us to predict AS and biliary leaks, while the risk of NAS and post-transplant cholangiopathy could not be investigated. Finally, BC arise from multifactorial causes including ischemic injury, immune responses, and surgical factors, which spectral analysis alone may not comprehensively capture. Larger datasets and subsequent in-depth analysis may allow for more precise discrimination between different etiologies of BC in the future. Addressing these limitations will be essential for future research to realize the full potential of HSI in LT.

Nevertheless, this first attempt to apply the potential of HSI to extract physical tissue properties should offer the basis for a future dynamic preoperative prediction model of BC in the clinical context of NLMP and LT. This approach may become an innovative, non-invasive preoperative tool for surgeons in the clinical routine [5].

## 5. Conclusions

The results of this study revealed significant differences in the spectra of BD data depending on their position (distal or proximal), the presence of complications, and whether an organ had been transplanted or not. While these findings demonstrate the potential of spectral analysis to capture clinically relevant differences, further studies with larger datasets will be needed to validate these results and to explore their integration into predictive algorithms that could ultimately support clinical decision-making.

## Figures and Tables

**Table 1:** Demographic data.

Paramter	Total (n = 7)
Donor and operative data	
Age (y) *	61 (51–68)
Gender	
Man	5 (71)
Woman	2 (29)
CIT (h) *	6 (5–8)
Cause of death	
Cerebrovascular	2 (29)
Circulatory	1 (14)
Trauma	2 (29)
Other	2 (29)
Donor Type	
ECD	6 (86)
DBD	6 (86)
DCD	1 (14)
NLMP indication	
Complex recipient	1 (14)
Marginal donor	3 (43)
Logistics	4 (57)
NLMP time (h) *	19 (11–21)
Total preservation time (h) *	22 (18–27)
Recipient data and postoperative outcome	
Age (y) *	59 (58–65)
Gender	
Man	5 (71)
Woman	2 (29)
BMI (kg/m^2^) *	25 (23–30)
MELD *	16 (13–19)
BAR score *	7 (5–8)
BAR score ≥ 8	2 (29)
Total hospital stay (d) *	16 (15–26)
ICU stay (d) *	3 (3–5)
Early allograft dysfunction	3 (43)
Clavien Dindo ≥ 3	5 (71)
30—days readmission rate (unplanned)	1 (14)
Biliary complications	2 (29)
≤30 d	2 (29)
>30 d	1 (14)
Biliary leakage	1 (14)
Anastomotic stricture	2 (29)
Arterial complication	3 (43)
Acute rejection	1 (14)
Infectious complication	5 (71)
Mortality rate	0 (0)
Re-transplantation rate	0 (0)

Values in parentheses are percentages unless indicated otherwise; * values are median (i.q.r.). BMI, Body Mass Index; ICU, Intensive care unit; CIT, Cold ischemia time; ECD, Extended criteria donor; DBD, Donation after brain death; DCD, Donation after cardiac death; DRI, Donor Risk Index; NLMP, Normothermic machine perfusion; MELD, Model for End-Stage Liver Disease; BAR, Balance of Risk Score.

## Data Availability

The data supporting this study’s findings are available from the corresponding authors upon reasonable request.

## Abbreviations

ALT	Alanine aminotransferase
AS	Anastomotic stricture
AST	Aspartate aminotransferase
BAR	Balance of risk score
BD	Bile duct
BC	Biliary complications
CIT	Cold ischemia time
DCD	Donation after cardiac death
DBD	Donation after brain death
EAD	Early allograft dysfunction
ECD	Extended criteria donors
ERCP	Endoscopic retrograde cholangiopancreatography
HSI	Hyperspectral imaging
HTK	Histidine-tryptophan-ketoglutarate
ICU	Intensive care unit
IGL-1	Institut Georges Lopez
IQR	Interquartile range
IRI	Ischemia–reperfusion injury
LT	Liver transplantation
MELD	Model for end-stage liver disease
MRCP	Magnetic resonance cholangiopancreatography
NAS	Non-anastomotic stricture
NLMP	Normothermic liver machine perfusion
PNF	Primary non-function
ROI	Region of interest
